# Parents’ reasons for nonadherence to referral to follow-up eye care for schoolchildren who failed school-based vision screening in Cross River State, Nigeria—A descriptive qualitative study

**DOI:** 10.1371/journal.pone.0259309

**Published:** 2021-11-18

**Authors:** Lynne Lohfeld, Christine Graham, Anne Effiom Ebri, Nathan Congdon, Ving Fai Chan

**Affiliations:** 1 Centre for Public Health, School of Medicine, Dentistry and Biological Sciences, Queen’s University Belfast, Northern Ireland, United Kingdom; 2 Clinical and Epidemiological Eye Research Center, Wenzhou Medical University, Wenzhou, China; 3 Nelson R. Mandela School of Medicine, KwaZulu Natal, Durban, South Africa; 4 Brien Holden Vision Institute Foundation (Africa) Trust, Durban, South Africa; 5 Zhongshan Ophthalmic Centre, Sun Yat-sen University, Guangzhou, China; 6 Orbis International, New York, NY, United States of America; 7 College of Health Sciences, University KwaZulu Natal, Durban, South Africa; Cairo University Kasr Alainy Faculty of Medicine, EGYPT

## Abstract

**Background:**

Uncorrected refractive error (URE) is a major cause of vision impairment in children worldwide. Cases are often detected through a school-based vision screening program and then treated in a follow-up appointment. This requires parents or guardians (‘parents’) to adhere to referrals for the eye exam and care plan. We aim to understand the reasons for parents’ referral non-adherence in Cross River State, Nigeria, using qualitative methods.

**Methods:**

Ten focus groups were held with parents who had not adhered to the referral for a follow-up eye examination. Participants were recruited with help from staff in schools hosting the vision screening programme. Interviews were conducted using a semi-structured interview guide, audio taped and transcribed verbatim. After identifying relevant quotes, the researchers labelled each one with a descriptive code/subcode label. Then they clustered the data into categories and overarching themes.

**Results:**

Forty-four parents participated in 10 focus group discussions with 28 women and 16 men. Three themes and participated in the focus group discussions with 28 women (63%). Twelve themes were generated. The three megathemes were Modifiable Factors (with 4 themes), Contextual Factors (with 6 themes), and Recommendations (with 2 themes).

**Conclusion:**

Participants identified modifiable barriers that make it difficult for parents to adhere to a referral for a follow-up eye exam. These include not believing their child has a vision problem or the screening test, and issues with the referral letter. They also described important contextual factors such as poverty, logistical problems, parental attitudes towards their children and beliefs about appropriate care. Many of these issues could be addressed by following their recommendation to educate the public on the importance of child eye care and correct parents’ misconceptions. These themes will be used by the Nigerian government to enhance and scale up its child eye health programme.

## Introduction

Approximately 17.5 million children suffer from vision impairment and 1.4 million are blind globally, with most of them living in low- and middle-income countries (LMICs) [1–3]. Uncorrected refractive errors are the leading cause of childhood vision impairment in much of the world, including Africa [4,5], with myopia the most common type of refractive error [6]. Nigeria, where the present study was conducted, is home to nearly one-quarter of Africa’s visually impaired or blind children [7], and has a refractive error prevalence rate of 5–8% [8].

Visual impairment greatly affects people’s health and quality of life [9], and is among the 10 most frequent causes of disability in Africa [10]. Uncorrected refractive errors are known to have a major economic impact on households and nations [11], causing an estimated annual global loss of US $269 billion as of 2009 [12]. For these reasons, childhood blindness has been identified as a high-priority issue by the “World Health Organization’s VISION 2020: Right to Sight” program [13].

Because refractive errors are asymptomatic, it is difficult for parents to recognize that an affected child is not seeing well. To address this issue, the Nigerian Government ran its Comprehensive Child Eye Health in Nigeria (CCEHiN) programme from 2017 to 2020 in 11 states, including Cross River State. According to the midterm report 656,554 schoolchildren were screened and 10,872 pairs of spectacles were supplied.

Currently the Nigerian Ministry of Health is pilot testing an eyeglasses cross-subsidisation component to the program in Cross River State, where the current study was conducted. Under this plan, some of the profits from selling more expensive spectacles are used to help underwrite the cost of other frames for low-resource households.

Many child eye care programmes run school vision screening with referral to a professional eye examination for children who fail the screen. Success depends on whether parents and guardians (‘parents’) adhere to the referral [14–16]. To increase adherence depends on understanding parental perception about children’s vision problems and knowledge of the eye care program [17,18]. To date, there has been relatively little work done on this issue in Nigeria [19]. To fill this knowledge gap, we conducted a study to explore reasons for referral nonadherence. This study is part of a larger research project, described elsewhere [20], to help the Nigerian government improve and scale up its child eye care programme.

## Materials and methods

### Study design and setting

This is a descriptive qualitative study [21] consisted of focus group interviews with parents of children who failed a vision test (<6/12 in either eye) and were not seen by an eye care professional. The study was conducted in these four areas of Cross River state: include Calabar (the capital city), Ugep (a town located 123 km or 77 miles from Calabar), Ikom (217 km/ 134 miles) and Ogoja (300 km/ 186 miles).

### Sample and recruitment

All participants were living in one of the four study sites, and parent of a child who had failed the school vision screening and not attended a follow-up eye exam. We then applied a criterion based purposive sampling frame to identify eligible parents based on their child’s school level (primary versus secondary) and 2) and type (‘urban’ = a large school located near the town centre versus ‘rural’ = a smaller school located further away). We intended to conduct eight to 12 focus groups (FGs) in order to reach saturation [22] but reached that point after 10 FGs.

Parents were recruited with help from classroom teachers who maintained a list of eligible parents. They gave their list to the research officer who contacted families by phone to confirm eligibility, explain the study and invite them to join a FG. About two weeks before the scheduled interview, the research officer called again to reconfirm interest, obtain oral consent and schedule transport to the interview site. Before each FG, parents provided written informed consent and were asked again if they had adhered to the referral for a follow-up eye exam for their child.

### Data collection

The interview guide was designed to elicit information about parents’ views on the importance of good vision for children, their knowledge of the CCEHiN programme, barriers and facilitators associated with attending a follow-up exam and recommendations to improve the program [23]. It was developed by two public health optometrists (VFC and AE), a medical anthropologist (LL) and a social anthropologist (CG) with input from staff at participating eye clinics. The topics, questions and probes in the guide are shown in Table 1.

**Table 1:** Focus group interview guide.

Topic	Questions and probes
Ice-breaker	What is your favourite meal?
Knowledge of eye care programme	How many of you know that your child’s school offered free vision screening, or an eye test was conducted on your child? • What were you told about the results of the eye test? • What were you told by your child’s teacher?
	After your child had their vision tested at school, what were you told you should do about your child’s eye health?
Importance of good vision for child	What do you think that might mean for your child’s health and wellbeing? • What about now? • What about in the future?
	Why do you think further testing was recommended for your child’s eyes?
Facilitators and barriers to adhering to referral for a follow-up eye exam	We know some parents or guardians of children who are referred for further examination at the eye clinic choose not to do so. What do you think are the main reasons for this? • Who in the household generally makes such decisions?
	Why do you think some families choose to bring their children in for an eye examination? • Who in the household generally makes such decisions?
Key messages, including recommendations to improve the CCEHiN programme	What message would you like me to bring back to the other researchers on the team?
	Thinking back to everything we discussed today, what would be your key message? • What about key messages for parents?

Four fieldworkers, working as two facilitator/note-taker teams, led each FG after completing a three-day training programme offered by VFC and AE in July 2019. This included practice interviews with two groups of parents (pilot data not part of the dataset). The fieldworkers had at least five years’ experience conducting community research; three of them were practising optometrists with O.D. (Doctor of Optometry) qualifications; and one note-taker had a college education. They facilitated the FGs in English or pidgin (local dialect) from 15 October to 30 November 2019. Each session lasted 90–120 minutes and was audio recorded with participants’ permission. The facilitators created verbatim written documents (transcripts) from the recordings, comparing the audio and written versions to identify and correct errors.

### Data analysis

Qualitative Content Analysis [24,25] was carried out by LL, VFC and AE. They first read each transcript to gain an overall sense of the data. They found no significant differences by school level or location and so included all transcripts in a single data set. They shortened each transcript by removing all but the relevant statements which they labelled with an anonymous ID (e.g., ‘FG3-M1’ refers to a quote by the first male participant in FG #3). They created a set of tables, one for each topic, to be populated by relevant quotes, a brief summary of the quote’s meaning, and a two-part descriptive label (code/subcode). Each transcript was double coded separately by two analysts who compared their entries on the topic tables. Any discrepancies were discussed until they reached consensus. They made a clean copy of the tables and entered the codes plus a brief definition and exemplary quote into a codebook. This process was repeated for all 10 transcripts. A final table was created with all the coded data from each transcript. Finally, they produced rank-ordered frequency tables for each topic. Key findings were both highly salient (mostly frequently used codes) and relevant (mentioned in at least half of the FGs) by theme.

To increase rigour of the data, no more than eight participants were planned per FG so that the facilitators could easily capture all participant views. The fieldworkers were trained in interviewing techniques and use of the facilitation guide, spent time building rapport with participants by explaining the process, asking an ice-breaker question and offering light refreshments. All interviews were conducted in an empty classroom in the local school for privacy.

To distinguish between researcher and participant views, quotes appear in italics and are labelled with a source ID. The analysts lightly edited quotes to increase readability while retaining their original meaning. Ellipses (…) show where words were removed and text in square brackets indicate where they have been added.

The study was approved by the Medical Research and Ethics Committees at Queen’s University Belfast (Pre FREC Ref 19.24v3) and the Cross River State Ministry of Health’s Health Research and Ethics Committee (CRS/MH/HREH/019/ Vol.V1/175). All participants gave oral consent upon joining the study and re-confirmed it in writing before being interviewed. All procedures were carried out in accordance with the Helsinki Declaration.

To ensure high-quality findings, the researchers followed a well-designed research protocol, pilot tested the interview guide, carefully trained the data collectors, maintained an audit trail, had a multidisciplinary team that included an experienced qualitative researcher analyse the data (researcher triangulation), supported all findings with evidence (quotes) and used criteria from the Standards for Reporting Qualitative Research (SRQR) criteria to evaluate the research process and methods [26].

## Results and discussion

### Demographic profiles of study participants

Forty-four parents took part in 10 focus groups; 28 (63%) of them were female. Participants ranged in age from 23–61 years (mean 41.3 + 7.8 years) and had from one to seven children (mean number 3.6 + 1.3). Most participants were civil servants or small-scale businesspeople. A few of them were farmers, clergy, housewives or unemployed. The focus groups ranged in size from two to eight members (mean = 4.4). All but one group consisted of both female and male members (Table 2). The occupational category ‘civil servant’ also includes health worker, public servant, social worker and teacher. The category ‘businessperson’ include lottery ticket seller, seamstress and trader. ‘Other’ refers to farmers, housewies, clergymen and the unemployed.

**Table 2:** Characteristics of focus group participants (n = 44).

FG ID	Female: Male	Age (years)	# Children	Occupations
1	3:1	30–39 (x3), 50–59 (x1)	2–3 (x2), 4+ (x2)	civil servant (x2), businessperson x2)
2	3:1	30–39 (x1), 40–49 (x2), 50–59 (x1)	2–3 (x4)	civil servant (x3), businessperson (x1)
3	2:2	30–39 (x1), 40–49 (x2), 50–59 (x1)	4+ (x4)	businessperson (x4)
4	3:1	30–39 (x3), 40–49 (x1)	2–3 (x3), 4+ (x1)	businessperson (x2), civil servant (x2)
5	1:1	30–39 (x1), 50–59 (x1)	4+ (x2)	businessperson (x1), civil servant (x1)
6	2:3	30–39 (x2), 40–49 (x2), 50–59 (x1)	2–3 (x1), 4+ (x5)	other (x1), civil servant (x4)
7	3:1	30–39 (x1), 40–49 (x2), 50–59 (x1)	2–3 (x3), 4+ (x1)	businessperson (x1), civil servant (x2), other (x1)
8	4:2	30–39 (x3), 40–49 (x1), 50–59 (x2)	2–3 (x3), 4+ (x3)	businessperson (x1), civil servant (x1), other (x4)
9	3:0	20–29 (x1), 30–39 (x2)	2–3 (x2), 4+ (x1)	other (x3)
10	4:4	30–39 (x4), 40–49 9x3), 60–69 (x1)	1 (x1), 4+ (x7)	businessperson (x2), civil servant (x3), other (x3)

### Themes, categories and codes

Within each theme, key categories that are both highly salient (i.e. most frequent categories per topic) and relevant (discussed in at least half of all focus groups) were identified. Each category is presented in Table 3 below; key categories are underlined. For the purpose of this paper, beliefs are views people hold as true or false without proof and attitudes are their general predisposition to view something as negative or positive. In the data we identified three megathemes and 12 themes (Table 3).

**Table 3:** Megathemes and themes derived from 10 focus group transcripts.

Megatheme	Themes
1) Modifiable barriers	Theme 1–1: Parental belief there is no eye problem. Theme 1–2: Issues with the referral letter. Theme 1–3: Parental belief they must pay for follow-up eye care. Theme 1–4: Parental beliefs about eyeglasses.
2) Contextual factors	Theme 2–1: Parental or household situation Theme 2–2: Logistical problems Theme 2–3: Parental attitudes Theme 2–4: Parental beliefs about care Theme 2–5: Illiteracy. Theme 2–6: Hospital-related issues
3)Recommendations	Theme 3–1: educate the public about the importance of child eye care. Theme 3–2: correct parental misconceptions about the eye care programme.

### Megatheme 1: modifiable barriers

#### Theme 1–1: Parental belief there is no eye problem

When asked why parents do not bring their child to a follow-up exam, a key reason was the belief that there was no problem with the child’s eyes or vision. As one mother explained, *“I felt there was nothing wrong with his eyes because he’s never complained to me* [so] *I didn’t take it serious”* (FG4-F1). Another mother shared a similar view: “[It’s] *disbelief …* —*not believing there’s a problem*. *They will say there’s no need for further testing because there’s no problem”* (FG9-F1). Still another respondents labelled this as ignorance:

*“Some ignorant parents may think that it is just ‘eehh’;* [indicating not very important with a shrug]. *It’s ignorance on the part of the parents; they’re unaware of what’s going on”* (FG1-M1).

Some parents clearly stated they generally disregard what young children say: “[With a young child] y*ou will not even take anything serious you will relax*. *That’s the reason”* (FG8-F1).

Another reason parents do not take action about a problem is because it does not seem serious:

*“Since my child wasn’t complaining about a physical problem*, *I felt it may not be important for me to go there*. *There wasn’t any eye drooping and he* [referring to her child] *was going about with his normal business so I felt it isn’t that serious and I overlooked it”* (FG1-F1).

#### Theme 1–2: Issues with the referral letter

A second key reason why parents do not bring eligible children for a follow-up eye exam is that the only information about the eye care programme is a referral letter that all children who fail the screening test are asked to bring home:

*“What made me not go* [to the eye clinic] *with the child is that I didn’t receive any note from them* [the school]” (FG10-F3).

In some cases the child may bring the letter home but not explain its contents to the parents. In other cases, parents reported that the referral letter did not look official so they ignored it:

“*I didn’t get confirmation from the school* authority to go for further test; that’s why I couldn’t go for the eye test” (FG10-M4).

This statement should be understood in the context of Nigeria:

*“If something isn’t from the government … people will be afraid*. *Some people believe only government officials are supposed to go into schools for immunization or eye treatments*, *or anything to do with children’s health”* (FG4-M1).

#### Theme 1–3: Parental belief they must pay for follow-up eye care

Many parents had the misperception there was a cost associated with clinic-based eye exams. As one mother noted, many parents *“think it’ll cost money*, *so they don’t want to go because of the possible financial costs”* (FG5-F1). In part, this is because hospital care can be financially out of reach for many people and the CCEiN eye clinics are hospital-based:

“*As far as Nigeria is concerned … less privileged people cannot go to hospital … they know that the nurses and the doctor won’t pay attention to them if they don’t bring money*. *First*, *they ask you to buy* [a patient registration] *card; then they prescribe drugs and ask you to buy* [them]. *They won’t even start treating you without you paying money*!*”* (FG2-M1)

#### Theme 1–4: Parental beliefs about eyeglasses

A few people also expressed negative views about eyeglasses, either because they believed *“glasses are for adults and not children”* (FG10-F2) or *“wearing glasses can negatively affect one’s eyes”* (FG10-M3).

### Megatheme 2: Contextual factors

Although contextual factors lie outside the purview of a health care program, they can affect its acceptability, uptake, scalability and sustainability. Therefore, it is important to understand other factors that are barriers to seeking follow-up eye care for children. Key among these factors are poverty, unhelpful attitudes towards children or beliefs about healthcare.

#### Theme 2–1: Parental or household situation

A major topic of discussion in each FG was the impact of deep poverty in each of the study sites. One woman bluntly stated, *“poverty is an understatement”* (FG10-F1). Another woman described how poverty resulted in her not adhering the referral: *“At the time there wasn’t even food for us to eat … that’s why we didn’t go* [to the eye clinic]*”* (FG7-F2). This was a common view, expressed by another FG participant:

*“Simply put*, *a lot of our people are poor*, *and poverty affects our reasoning*. *You run around looking for things to eat and only later think about other problems that will bring them sorrow later*” (FG5-M1).

In other groups, participants discussed both the need to postpone healthcare visits and the lack of affordable transport as barriers to attending follow-up eye exams.

#### Theme 2–2: Logistical problems

A sizeable number of comments were made about time- and work-related factors as important reasons why parents cannot accompany their child to an eye clinic: *“Money is not the only reason; some parents are too busy”* (FG9-F1). Another parent echoed this view, stating, *“It all boils down to time*. *Some parents have very tight schedules”* (FG6-M3). This is closely linked to the inability to attend scheduled appointments:

*“We know that when the time comes to do an eye check-up for children*, *some people won’t go*. *They’ll have an appointment scheduled but might not be available* [at that time].*”* (FG10-F4)

Often this is because parents cannot take time off work: *“My work didn’t permit me to—it didn’t give me free time to take him there”* (FG4-F1).

#### Theme 2–3: Parental attitudes

Participants also used generally negative terms to describe parents who do not bring their children for care, indicating they are *‘careless’*, *‘negligent’*, *‘forgetful*’ or *‘disinterested in their children’*. As one mother noted, *“If a parent refuses to take their child for further care in this kind of situation*, *it’s probably due to carelessness”* (FG5-F1).

In other instances, parents said *“for some*, *it’s due to negligence”* (FG4-M1) or forgetfulness:

*“It escaped my memory*. *Even when I was thinking about it today*, [I was wondering], *will I be available to go*? *But before the day* [of the appointment], *it escaped my memory”* (FG6-F2).

There was also a widespread perception that some parents do not take proper care of their children: *“Some parents only give birth to their children*, *but don’t take care of them the way they’re supposed to*.*”* (FG8-M2) Some participants went so far as to state there are parents who neglect their children and still expect to be taken care of:

*“Ehh*, *some children suffer a lot*. *Their parents are only interested in their children helping them*, *not the parents helping their children… they don’t even eat well*!*”* (FG5-M1).

In other cases, it was thought that some parents are just not interested in their children’s welfare:

*“Some of them just take things for granted*. *Most of them actually have the money to take the child to the hospital but they just take everything for granted and just leave it alone*.*”* (FG9-F1)

This was also linked to having a large family: “[Some parents] *don’t value their children because they have so many”* (FG5-F1). In other cases, it was attributed to a lack of empathy on the part of the parents: *“Some people will be the end of their families*. *They don’t feel pain from things that don’t happen to them”* (FG2-M1). In some instances, this was because the caretaker was not a biological parent:

*“Some guardians don’t take their children there* [referring to hospital] … *because they’re not the person who gave birth to those children… they won’t spend their money on the child because it’s not their biological child… They’d prefer to leave such children like that and allow their eyes to get bad*, *which is very bad”* (FG8-M2).

It is important to determine who is the usual decision-maker regarding children’s health. Although parents often stated that it is the father who decides, probing revealed both parents often jointly make decisions affecting a child’s health and the household budget. However, they also admitted that the father is the ultimate authority. As one woman stated, *“In a family if your husband says ‘No’* … *the child won’t go to the hospital”* (FG9-F1).

#### Theme 2–4: Parental beliefs about care

Participants provided several examples of parents’ beliefs about hospitals that can prevent them from seeking care for their child. This includes a belief that *“going to the hospital is taboo”* or that the hospital is only *“for big people* [or] *people that inherit sickness”* (FG2-F3). Another belief is that once a health problem is named, the person will become ill with that condition. One father explained that situation as follows:

*“A main reason why some people don’t go to the hospital is they don’t think they’re sick*. *But once they go to the hospital and they [the hospital staff] tell them they have a disease*, *then they become sick… they believe that if someone mentions a sickness of some kind*, *they will get it so that makes some people afraid to go to the hospital”* (FG5-M1).

In another FG, one of the participants described knowing someone whose vision worsened after receiving treatment. He ended his story by stating that *“some* [parents] *could be afraid of further damaging their children’s sight*.*”* (FG10-M3)

In Nigeria, spiritual beliefs strongly shape health-seeking behaviors. It is very common for people to believe in the power of prayer as well as in allopathic or modern biomedicine, choosing to pray before seeking medical care:

*“Some parents are like me*. *When something happens*, *I first will handle the spiritual aspects before getting medical attention because I know there are some cases that were resolved that like in the past… some of us believe that the spirit controls the physical*. *It’s not that we don’t believe in the power of modern medicine*, *but sometimes it* [the problem] *can be a spiritual attack”* (FG10-M4).

In many communities, several people attend Apostolic churches that forbid the use of modern medicine. Members of one FG acknowledged this, stating *“It may be because of their religion*. *Like the Apostolic faith*. *They tell you*, *‘Don’t use Western medicine’”* (FG2-F1). Many Pentecostal or charismatic churches also do not endorse the use of allopathic medicine and instead exhort members to rely on the power of prayer or laying on of hands to cure health problems. As another participant explained, *“Some of them* [parents] *don’t believe in medical treatment*, [because of] *their orthodox church”* (FG1-M1). Instead, they expect that divine intervention will heal a person. Another reason for avoiding biomedical facilities is a belief that health problems are due to witchcraft, which is overcome through prayer:

*“Sometimes it’s not a financial issue* [that prevents people from attending an eye clinic] *but* [a belief that] *a problem is due to attack* [witchcraft]. *Then we need to fasten up in prayers* [seriously pray]” (FG10-F4).

Some families rely traditional remedies to treat eye or vision problems:

*“I was taking care of a blind man and asked him*, *‘How did you become blind*?*’ He said*, *‘It’s because of measles*.*’ He had measles when he was young and his parents put drops of a traditional remedy in his eyes*. *‘Why*?*’*, *I asked him*. *He said*, *‘They didn’t know they should’ve taken me to the hospital’*.*”* (FG10-F1)

#### Theme 2–5: Illiteracy

The lack of proactive care of children’s vision was also attributed to low levels of literacy or health literacy. One man explained, *“We still have a lot of illiteracy in town”* (FG4-M1). This presents a problem when schoolchildren who fail the screening bring home the referral letter for a follow-up exam:

*“They* [referring to his children] *bring home letter for their mother*, *who can’t read*. *And she says*, *‘Give me the letter and when your father comes* [home] *I’ll give it to him to read*.*’ By the time the father comes home*, *she’s forgotten so she doesn’t give the letter to him*!*”* (FG7-M1).

#### Theme 2–6: Hospital-related issues

There were also complaints about hospitals which could result in parents not adhering to referrals to a hospital-based eye clinic. Two key issues were negative attitudes from staff: *“Doctors have to show sympathy*. *Some doctors*, *they don’t have that human sympathy”* (FG2-F1) and long wait times to be seen by a doctor:

*“You go there* [to the hospital] *but don’t get to see the doctor… That’s stressful for parents because they have things to do at home rather than going to the hospital and wasting the whole day* [waiting]” (FG2-F1).

### Megatheme 3: Participant recommendations

The two recommendations made were to educate people about the importance of child eye care, and to correct parental misperceptions about the CCEHiN programme, such as thinking they would have to pay for the appointment or that the healthcare team would return to the school to provide follow-up care at a later date.

#### Theme 3–1: educate people about the importance of child eye care

There was also some discussion on how to increase awareness and acceptance of the child eye care programme in Cross River State. A lack of knowledge or information was something that participants thought the CCEHiN programme could address: *“I think it* [not seeking follow-on care] *may be due to the lack of information … a parent may not be informed on how serious the issue of that child is”* (FG4-F1).

In some cases families are uninformed because they lack access to information: *“They* [referring to poor families] *may not have a radio or a television …* [which is]*where they would hear information like this”* (FG1-F2).

#### Theme 3–2: correct parental misconceptions about the eye care programme

The other recommendation, to increase awareness and uptake of the eye programme, would be to address common misconceptions, such as thinking that both vision screening tests and follow-up care are provided at local schools. As one father explained, *“I didn’t go* [to the clinic] *because* [I thought] *the team will come back to the school for further care”* (FG10-M4). A woman explained this was why she did not bring her children for a follow-up exam:

*“My children brought the referral letters to me*, *but I didn’t follow their advice*. *I followed up with* [an appointment at the] *General Hospital where my doctor promised me that the people* [offering eye care] *will come back* [to the school] *by September*. *So*, *I let the children wait*.*”* (FG10-F1).

Some parents also noted it is important to ensure that parents are given an official referral letter because *“some people believe only government officials are supposed to go into schools for immunisation or eye treatments–anything to do with children’s health”* (FG4-M1).

## Discussion

The link between good vision in children and their academic performance is well documented. Often children with poor vision have difficulties reading and focusing on academic tasks. Because most of what children learn is through visual processing, children with uncorrected vision problems cannot learn effectively. Untreated vision problems can also affect children’s emotional, neurologic and physical development.

Early diagnosis and treatment of vision problems can prevent irreversible damage. An important first step is to detect near- and far-sightedness, amblyopia and strabismus, as well as colour blindness and depth perception is through school screening programmes. However, the way to diagnose and treat vision problems for children failing a screening test is to have a follow-up professional eye exam. Often vision screening is done through schools. Children who fail the screen are referred for the eye exam and are expected to bring the referral letter home to their parents.

There are several reasons why parents choose to be nonadherent to referrals for follow-up eye examinations. First, some parents are not aware of the school vision screening results. Although this was not a prevalent reason in our study, other researchers have found this is an important barrier. This includes the study by Frazier et al. [27] in Alabama where the main reason for not attending an eye examination was having lost or never received the referral letter. Su et al. [28] similarly reported that the main reason eligible screened schoolchildren in New Haven, Connecticut, had not attended a follow-up examination was their parents were not aware of the screening results. These findings are also echoed in a pilot study by Noma and colleagues [29] in Sao Paulo, Brazil. Even with free eye exams on weekends, free spectacles and transportation to clinics, 41% of the eligible children did not attend a follow-up exam after the referral letter. The main reason parents gave for this is they did not know about the referral.

This fits well with an important finding from our study, where several nonadherent parents in our focus groups explained they had not booked an appointment because eligible screened schoolchildren were expected to bring the referral letter home. In some cases, the letter was lost or the child did not explain its significance to the parents. To address the problem of parents not being informed about the need for an eye examination, Yawn et al. [30] studied the impact of providing multiple referral letters to parents of eligible children in Rochester, Minnesota. Despite several reminders, the average lag time between the failed vision screen and a follow-up examination was about two years and even longer for younger schoolchildren.

Another barrier to attending professional eye exams is parents’ understanding and perceptions about eye diseases in their children [31,32]. Broadly speaking, this is a major barrier to utilizing a wide range of medical services [33–36]. Studies to identify these views include the work of Frazier et al. [27] with Hispanic parents in Alabama, Ramai et al.’s [17] comparative study in Ghana, Honduras and India, Tjam et al.’s [37] research with nonadherent parents in Rotterdam, the Netherlands, and Yawn’s [30] study in Rochester, Minnesota. In these and other studies, nonadherent parents reported they would only act if their child’s symptoms were both noticeable (e.g. red eyes, squinting, tearing and discharge, pain or child rubbing their eyes) plus severe. These results echo those from studies by Kovai et al., [33] Ebeigbe [19] and Amiebenomo et al. [38] in Nigeria. We also found many nonadherent parents reportedly thought their child’s vision was fine and so disbelieved screening results or ignored them. Some researchers note this has resulted in delayed appointments for up to four years while parents waited for the condition to become serious [16].

In our study we identified other reasons for nonadherence to referral for a follow-up eye exam including economic factors, logistical issues, family interference, issues with the referral letter and not knowing about the programme. Affordability is one of the criteria used to examine utilisation rates of eye care services. This is influenced by both income levels and costs, which should include the price of service or treatment (e.g., spectacles), out-of-pocket costs (e.g., for travel) and opportunity costs such as lost time at work [6] or long waits at a clinic [19]. Having insurance may offset some costs, but usually not all. [38] Economic barriers are linked to referral nonadherence in Nigeria [19,38], South Africa [39], Zimbabwe [40] and India [41].

Logistical issues include finding time to schedule or bring the child to an eye clinic. Several of our participants reported having to take time off work is a barrier to their adhering to the referral, as well as time spent waiting at hospital clinics. This was also reported in other studies [19,38,42]. Chu and colleagues [43] conducted a study in California in which they tested whether free eye exams on school premises would increase adherence and found only 51% of children came for the professional exam. This suggests that factors other than financial or logistical barriers may affect adherence rates.

Parental attitudes, sometimes referred to as family interference, can also result in referral nonadherence. This situation exists when decision-makers in the household or family disagree about bringing a child for an eye examination. This was documented in another study in Nigeria [19] as well as in our study. Participants frequently stated that the husband makes the decision about bringing a child to the eye clinic. However, probing revealed that frequently parents would discuss and come to agreement about this issue, even though the father could ultimately decide against the exam. This situation is common in Africa, where groups of influential people will jointly decide about healthcare expenditures and treatment options [44]. This also occurs in the US [45] and other high-income countries.

Another barrier to attending follow-up examinations in our study was lack of awareness about the eye care program, which was also noted in a review of interventions to improve school-based eye care services in LMICs [46]. We also discovered that many parents did not believe the referral letter was official and therefore credible because it was not from an official document from the government. This is similar to Frazier’s [27] finding that some nonadherent parents admit they are inattentive to school documentation.

One of our main findings is that parents often were unaware of the referral and so did not bring their child to for a professional exam. This suggests there is a need for improved communication between school or programme staff and parents. One strategy is to not rely on children to bring letters home and instead mailing them to parents. Telephone calls can effectively be used to inform and motivate people towards positive action [27], particularly when addressing perceptual barriers [43]. To increase referral adherence, it would be important to teach families to pay attention to the signs of uncorrected refractive error and the potential damage it can cause if not addressed. It is helpful if policymakers are involved in designing effective communication interventions to correct important misconceptions and long-held beliefs parents have about their children’s eye health [19].

Another key finding in our study was the importance participants placed on educating the public and correcting parents’ misperceptions about child eye health. This has been successfully done in other communities by focusing on the importance of regular screening and follow-up exams, prevention, and not overlooking refractive errors in children, particularly in school-aged (as opposed to younger) children [47,48]. Such messages can be disseminated via culturally appropriate posters and flyers [49,50], radio and TV programmes, billboards, posters, magazines, newspapers [51]. New technologies (internet, mobile phones, personal digital assistants) may be effective but more research is needed to determine their impact on child eye health. Successful campaigns target an episodic behavior and use multiple interventions [48], particularly material co-developed with local stakeholders [52] and are linked to available services.

### Strengths and limitations

There is growing acceptance that qualitative research in health fields is important to ensure that the voice of patients and the public are heard. However, there are relatively few reports on qualitative ophthalmologic studies appearing in print [53,54]. This is an important gap when promoting evidence-based policy and program development in ophthalmology [55,56]. Another strength is this study was designed with input from opthalmological service providers familiar with the Nigerian child eye care program, as well as investigators well versed in qualitative research methods.

One limitation of this study is the lack of sub-analyses by level of school (primary/secondary) or its location (‘urban’/‘rural’). This may have been an artefact of not collecting enough data for this analysis, or that similarities in underlying beliefs, attitudes and contextual factors in Cross-River State resulted in the relatively homogeneous findings. Further research should be done in communities with people of different ethnicity, language, culture and environment to determine how applicable findings from this study could be for other parts of the country or elsewhere in Sub-Saharan Africa. However, we have provided enough information that replication studies could be conducted elsewhere to identify the local patterns of parental beliefs and behaviours affecting their level of referral adherence for follow up professional eye examinations for children failing a school based vision screening.

## Conclusion

Factors affecting parents’ health-seeking behaviour play an important role in protecting children’s eye health. In this study we identified major barriers to referral adherence by parents. These ranged from local perceptions about children’s vision and appropriate care rather than the price of spectacles, which is often a major barrier for child eye care programmes. We also learned that parents strongly recommend the program mount educational campaigns in their communities. This information will be useful for the Nigerian government in its efforts to ensure more children with vision problems undergo a professional eye examination and, if needed, receive spectacles.
